# Role of ND10 nuclear bodies in the chromatin repression of HSV-1

**DOI:** 10.1186/s12985-016-0516-4

**Published:** 2016-04-05

**Authors:** Haidong Gu, Yi Zheng

**Affiliations:** Department of Biological Sciences, Wayne State University, 4117 Biological Science Building, 5047 Gullen Mall, Detroit, MI 48202 USA

**Keywords:** HSV-1, ICP0, ND10, Chromatin Remodeling, Viral Replication, Lytic infection, Latent infection

## Abstract

Herpes simplex virus (HSV) is a neurotropic virus that establishes lifelong latent infection in human ganglion sensory neurons. This unique life cycle necessitates an intimate relation between the host defenses and virus counteractions over the long course of infection. Two important aspects of host anti-viral defense, nuclear substructure restriction and epigenetic chromatin regulation, have been intensively studied in the recent years. Upon viral DNA entering the nucleus, components of discrete nuclear bodies termed nuclear domain 10 (ND10), converge at viral DNA and place restrictions on viral gene expression. Meanwhile the infected cell mobilizes its histones and histone-associated repressors to force the viral DNA into nucleosome-like structures and also represses viral transcription. Both anti-viral strategies are negated by various HSV countermeasures. One HSV gene transactivator, infected cell protein 0 (ICP0), is a key player in antagonizing both the ND10 restriction and chromatin repression. On one hand, ICP0 uses its E3 ubiquitin ligase activity to target major ND10 components for proteasome-dependent degradation and thereafter disrupts the ND10 nuclear bodies. On the other hand, ICP0 participates in de-repressing the HSV chromatin by changing histone composition or modification and therefore activates viral transcription. Involvement of a single viral protein in two seemingly different pathways suggests that there is coordination in host anti-viral defense mechanisms and also cooperation in viral counteraction strategies. In this review, we summarize recent advances in understanding the role of chromatin regulation and ND10 dynamics in both lytic and latent HSV infection. We focus on the new observations showing that ND10 nuclear bodies play a critical role in cellular chromatin regulation. We intend to find the connections between the two major anti-viral defense pathways, chromatin remodeling and ND10 structure, in order to achieve a better understanding of how host orchestrates a concerted defense and how HSV adapts with and overcomes the host immunity.

## Background

Herpes simplex virus (HSV) is a member of family *Herpesviridae*, genus *Simplexvirus*. After the primary infection at oral, genital or ocular mucosa, HSV establishes latency in ganglion sensory neurons. Periodically, HSV reactivates and transmits through symptomatic or asymptomatic shedding, causing an extensive spread of this virus worldwide. Over 70 % of the world adult population are seropositive for HSV, which marks HSV as one of the most prevalent opportunistic pathogens and the etiologic cause of a wide range of mild to severe herpetic diseases including cold sores, stromal keratitis and encephalitis.

Like all herpesviruses, the lifelong infection of HSV and its unique lytic-latent-lytic infection cycle warrant a close relation and an intricate balance between HSV and its host. In the case of HSV-1, the virus has a double-stranded DNA genome of 152 kb, encoding more than 84 viral proteins [1]. The large viral genome gives HSV adequate coding capacity to finely regulate the virus-host interaction over a long course of infection. For almost every aspect of human anti-viral defense systems, be it on a single cell level or on a whole body surveillance level, countermeasures have been found in the HSV-1 infection. These properties make HSV-1 an ideal model virus for understanding the relation between viral replication and host defense mechanisms.

Chromatin-regulated gene repressions and nuclear domain 10 (ND10)-associated anti-viral restrictions are two different cell responses interweaving together for a concerted host defense. HSV-1 employs multiple viral proteins, especially an α (immediate early) gene product called infected cell protein 0 (ICP0), to coordinate the counteractions against the two anti-viral defenses. This review focuses on the connections between the epigenetic regulation and ND10 dynamics. We will discuss the recent advances that shed light on the coordination of host defensive pathways and also the seemingly orchestrated viral countermeasures.

### Chromatin remodeling in lytic and latent HSV infection

In epigenetics, genes are activated or repressed through changing the status of histones or nucleotide modifications (for reviews, see references [2, 3]). Therefore, cell functions are modulated without changing the DNA sequences. Chromatin epigenetic regulation is one type of host autonomous anti-viral response that mostly targets against the DNA viruses. This intrinsic defense mechanism mobilizes cellular histones and histone associated complexes to quench viral transcription and replication.

#### Chromatin repression in lytic phase

Unlike other DNA viruses that can pack their virion genomes into minichromosomes to avoid being recognized as foreign DNA [4, 5], HSV-1 does not contain histones or histone-like proteins in the capsid [6]. Instead, early studies showed that HSV-1 had polyamines in the virion to neutralize the negative charges on viral DNA [7]. HSV-1 DNA is tightly confined within the capsid and endures a pressure of about 20 atmospheres [8]. This tremendous pressure drives a quick ejection of viral DNA into the cell nucleus upon infection [9]. The sudden injection of mostly naked viral DNA inevitably triggers an immediate alarm of foreign invasion. Indubitably the infected cell mobilizes all defensive forces and attempts to silence the viral DNA right away. One major host cell defense against the incoming viral DNA is the mobilization of histones and histone associated repressors to force the viral DNA into chromatin repression. Although the exact mechanism of how cells mobilize the histone pool is not clear, it has been shown that histones are more mobile after the HSV-1 infection [10–12]. At least partial or unstable nucleosomes are formed in the lytic infection, albeit unevenly across the viral genome [13, 14].

The inhibitory effects of chromatin formation on viral gene expression are reflected in several lines of evidence. First, HSV-1 DNA was found to associate with histone H3 as early as 1 h post infection [6]. Early in infection, more histone association was found at β (delayed early) and γ (late) gene promoters than that of α gene promoters [6, 15]. Viral proteins such as VP16 and ICP0 are responsible for the removal or remodeling of the histones, which leads to the activation of viral gene expression (see below). The second observation that chromatin formation represses HSV-1 expression is the fact that inhibitors targeting chromatin deactivating enzymes, such as histone deacetylases (HDACs) [16, 17], promoted viral gene expression and DNA replication for a recombinant HSV-1 containing a growth defect [18], indicating the significance of the reversal of histone deacetylation in lytic HSV-1 infection. The third evidence is the demonstration of functional interactions between HSV-1 proteins and chromatin repressors during the infection. For example, a nuclear repressor complex REST/CoREST/LSD1/HDAC was disrupted during the HSV-1 infection by ICP0, a viral gene transactivator that enhances downstream gene expression without any sequence specificity (for reviews, see [19, 20]), and then later in infection, CoREST and HDAC1 were translocated into the cytoplasm [21]. A dominant negative CoREST interfering the CoREST-HDAC1 interaction partially rescued viral replication in the absence of ICP0 [22], whereas the ICP0 mutant virus defective in CoREST binding showed a growth defect and failed to hyperacetylate the histone H3 and H4 bound to the quiescent DNA in a superinfection assay [23, 24]. ICP0 also interacts with class II HDACs and the interaction is responsible for the relief of HDAC5-mediated gene repression [25]. ICP0 has a comprehensive role in both histone removal and histone acetylation in lytic infection [26]. It is capable of promoting a two-step heterochromatin removal from the ICP8 promoter [27]. Interestingly, LSD1, the histone demethylase in the REST/CoREST/LSD1/HDAC complex, is required for early gene expression in both lytic and latent HSV-1 infection [28]. Since histone methylation status (mono-, di- or tri- methylation) plays different roles in gene activation or repression [29], how the inhibition of LSD1 changes histone methylation and how different methylation status regulates the initial HSV infection are not yet clear. Another viral protein, tegument protein VP16, is responsible for excluding histones from α gene promoters upon the viral DNA entry [15]. VP16 recruits host cell factor 1 (HCF-1) and Oct-1 to stimulate α promoter activity. This immediate counteraction against chromatin repression allows the expression of α genes, including ICP0 which further de-represses the HSV-1 chromatin on β and γ promoters [20, 26], and ensures a full blown infection. In consistent with these observations, newly synthesized viral DNA is not chromatinized and is well associated with RNA polymerase II and transcription factors [6, 30].

#### Chromatin repression in latent phase

In latent HSV infection, all viral genes are turned off except the latency-associated transcript (LAT), which is actively transcribed throughout the latency [31]. HSV DNA exists as episomes in latently infected sensory neurons [32, 33]. The viral DNA itself is not extensively methylated [34, 35], but the typical nucleosome-protected DNA pattern is readily observed for latent DNA in micrococcal nuclease assays, suggesting that latent viral DNA is packed into a nucleosomal structure like the host chromatin [36]. The viral latent chromatin is also regulated in a mechanism similar to that of the host chromatin. For example, the histone H3K9 and H3K14 at LAT promoter are hyperacetylated whereas they are hypoacetylated at lytic promoters, consistent with the fact that LAT is the only transcript made in latency while all other viral expressions are repressed [35]. Furthermore, injection of HDAC inhibitor into latently infected mice induces reactivation [37, 38], whereas application of an inhibitor specifically blocking the demethylation of the repressive marker H3K27me3 reduces reactivation in cultured neurons [39]. These findings suggest that changes of histone modification status may control the switch between latency and reactivation.

Interestingly, part of the LAT transcript is complementary to the C-terminal region of ICP0, the powerful heterochromatin remover that stimulates lytic infection. The promoters for LAT and ICP0 are only about 5 kb away [1]. To set apart the euchromatin of LAT promoter/enhancer region from the heterochromatin of ICP0 promoter region in the latent infection, HSV evolves to contain chromatin insulator CTCCC repeats within the LAT intron, which recruits the CTCF protein and marks the boundary between the euchromatin and heterochromatin of latent HSV DNA [40].

Although in latent infection HSV genome DNA is clearly packed into chromatin and HSV genes are fully regulated through the host epigenetic machineries, the processes of how chromatinization is initiated to establish latency and how chromatin repression is released to reactivate from latency are largely unknown. The expression of LAT is very important for the HSV-1 latency, which is reflected in two lines of evidence: (i) Deletion of LAT expression resulted in a reduction of histone H3K9me2 and H3K27me3, markers for inactive heterochromatin, and an increase of histone H3K4me2, a marker for active euchromatin, at the lytic promoters, indicating the participation of LAT in regulating chromatinization at lytic promoters of HSV-1 [41, 42]; and (ii) several microRNAs derived from the LAT region inhibited the expression of ICP4 and ICP0, the two major gene transactivators for lytic infection, suggesting that LAT also regulates lytic expression on posttranscriptional level [43]. More interestingly, the lack of LAT expression did not eliminate the presence of latent viral DNA in mouse ganglia [38, 41, 44], but greatly reduced the rate of spontaneous reactivation in infected animals [38, 45]. These results demonstrate that LAT expression is not required in latency establishment but it is essential for latency reactivation. Although it is still unclear how LAT is involved in stimulating the reactivation, it is conceivable to postulate that LAT may monitor the basal level expression of lytic genes by modulating the chromatin status at lytic promoters and by controlling the leaky transcription via microRNAs. Consequently, LAT works to fine-tune the balance between latency and reactivation.

Several chromatin repressor complexes are shown to be important for either latency establishment or latency reactivation. One of them is the aforementioned REST/CoREST/LSD1/HDAC complex. Specific inhibition of LSD1 blocked HSV-1 reactivation from latency [28, 46]. Another component from this complex, REST, plays a critical role in latency establishment. Overexpression of wild type REST in infected neuron caused a reduction in reactivation from the explanted ganglia [47], whereas overexpression of a dominant-negative REST capable of binding to DNA but not to the other complex components led to a failure in latency establishment [48]. A second repressor complex has been implicated in latency regulation is the polycomb group proteins including polycomb repressor complexes (PRC) 1 and 2 [49, 50]. PRC1 component Bmi1 and PRC2 component Suz12 were each found on lytic promoters during latency by two research groups [49, 50], but the results were not reconciled with each other. A recent report showed that histone phosphorylation by JNK pathway in the presence of repressive methylation also contributed to the initiation of latency reactivation [51]. How these different pathways cooperate to control the reactivation switch is still largely unknown.

### ND10 nuclear bodies in the restriction of HSV infection

ND10s, also known as PML (promyelocytic leukemia) nuclear bodies or PML oncogenic domains, are nuclear structures that are composed of over 150 constituents [52]. PML is the key organizer protein [53–55] for ND10 while many other ND10 components are only recruited upon specific stimulations (for reviews, see references [56]). ND10 is functionally promiscuous and has been implicated in numerous cell functions including gene regulation [57, 58], cell cycle arrest [59], apoptosis [60], DNA repair [61], oncogenesis [55, 62], and anti-viral defense [56]. The anti-viral effects of ND10 are initially suggested by the following lines of evidence: (i) Interferon (IFN) treatment increased the expression level of PML and Sp100 and also the number and size of ND10 bodies in treated cells [63, 64]. (ii) Disruption or distortion of the ND10 structure is a common theme for many viral infections. For example, major ND10 constituents were found degraded, which led to the dispersal of ND10 bodies, in HSV-1 and in HCMV (human cytomegalovirus) infections [65, 66], whereas ND10 deformation and reorganization were observed in adenovirus and papillomavirus infections [67, 68], respectively. (iii) PML knockout mice were found prone to infections [69]. Following an IFN treatment, PML-/- fibroblasts failed to curtail viral replication to the same extent as their PML+/+ counterparts [70, 71].

The relation between HSV-1 and ND10 during the lytic infection is undoubtedly an intimate one. Upon entry into the nucleus, HSV-1 viral DNA is first found in the vicinity of ND10 bodies [72]. Although it remains unclear whether this convergence of ND10 and viral DNA directly causes the modulation of viral DNA, it is quite evident that HSV-1 makes great efforts to destroy the ND10 structures. ICP0, the immediate early protein that promotes downstream viral expression, interacts dynamically with the ND10 nuclear bodies [73]. Upon synthesis, ICP0 is recruited to interact with ND10 [74]. Three proline-rich segments in the central region of ICP0 facilitate ICP0 to fuse with the ND10 bodies so that ICP0 can extensively comingle with the ND10 components [75]. More importantly, ICP0 contains a RING-type E3 ubiquitin ligase activity in its N-terminal region [20]. The extensive interaction between ICP0 and ND10 components triggers the proteasome-dependent degradation of two major organizers of ND10, PML and Sp100 [65]. The degradation of ND10 organizers leads to a subsequent dispersal of the ND10 bodies [76, 77]. The dispersal of ND10 components is a key event in HSV-1 replication. When ICP0 is deleted, or when the E3 ubiquitin ligase of ICP0 is mutated, or when ICP0 fails to enter ND10, ND10 persists at viral DNA and viral replication is greatly demolished, especially at low multiplicity of infection [73, 78, 79]. Conversely, if ND10 components such as PML, Sp100, Daxx and ATRX are depleted by siRNA knock-down, individually or in combination, viral replication is significantly enhanced in the absence of ICP0 [80–83]. Although the molecular mechanism of how ND10 regulates HSV replication is not completely clear, recent discoveries suggest that ND10 may inhibit HSV-1 expression, at least in part, through chromatin regulation.

### Interconnection between ND10 and epigenetic regulation

ND10 nuclear bodies are also called PML oncogenic domains because of the tumor suppressor function initially identified for PML. A t(15;17) chromosome translocation generates a PML-retinoic acid receptor α (PML-RARα) chimeric fusion, which acts as a dominant-negative PML to disrupt the ND10 structures. This is the etiological cause for acute promyelocytic leukemia (APL) [84]. All-*trans* retinoic acid (RA) treatment restores the ND10 nuclear bodies that are disrupted by the PML-RARα fusion and drives APL into remission [85].

The connection between chromatin remodeling and ND10 nuclear bodies has been proposed by many cell biologists and cancer biologists, based on a series of experimental and clinical observations. First, various types of histone modification enzymes, including acetyltransferases, deacetylases, and methyltransferases, have been found to accumulate at ND10 [86–88]. A direct physical interaction between PML and HDAC demonstrated by Wu et al [87] also showed that an inhibition on gene expression was caused by the PML-HDAC association. Furthermore, drugs inhibiting deacetylation by HDAC or demethylation by LSD1 promoted RA differentiation pathways via chromatin remodeling, which helped to differentiate leukemia blasts that are resistant to the RA-only treatment [89, 90]. The similar therapeutic effects obtained from HDAC or LSD1 inhibition, which restores the ND10 structure in APL patients, suggest that ND10 integrity and ND10 functions are regulated through chromatin remodeling.

The second major indication that ND10 is closely associated with chromatin regulation is the accumulation of histone chaperones such as HIRA, Asf1 and Daxx at ND10 [91, 92]. These chaperones participate in the assembly and disassembly of nucleosomes and regulate the incorporation of histone variants to reprogram the chromatin (for reviews, see references [93, 94]). The localization of histone chaperone proteins at ND10 suggests the involvement of ND10 in reassembling nucleosomes under various physiological conditions, such as cell senescence or DNA damage repair [91, 95].

The third observation supporting a close ND10-chromatin relation is the accumulation of numerous chromatin regulators at ND10 or their direct interactions with ND10 components. These regulators include general repressive proteins such as heterochromatin protein 1 (HP1) [96], corepressor N-CoR, Sin3A [97] and TIF1β [98], and general transcription activators such as CBP [99], STAT3 [100], Sp1 [101] and HIPK2 [102], just to name a few. The presence of these factors in the dynamic ND10 depends on the cell type and cell physiological status. The effects these factors may bring to the cell also vary for different genes at a given time. One interesting phenomenon stemmed from tethering reporter gene to ND10 [103]. When a SV40 promoter-driven luciferase was targeted to ND10 the transgene was repressed, but when a CMV promoter-driven luciferase was targeted to ND10 it was activated. Moreover, when ICP0 was co-expressed, the expression of both tethered luciferase minigenes was elevated [103]. These results suggest that (i) distinctive promoter sequences are modulated differently by the various ND10 components, and (ii) ICP0-targeted PML degradation and ND10 dispersal can obscure the DNA sequence specificity and put the DNA up for activation.

### Role of ND10 in regulating HSV chromatin during lytic and latent infection

As discussed in the previous sections, both chromatin repression and ND10 nuclear bodies are regarded as important parts of the host intrinsic anti-viral defense mechanisms [21–24, 80–83]. A few lines of evidence have shown that these two defenses interweave with each other. ND10 restricts viral replication, at least partially, through regulating the chromatin status of the HSV genome.

In latently infected neurons, the number of HSV-1 genome loci varies from neuron to neuron, suggesting the heterogeneity of latent infection [104]. In neurons containing a single HSV-1 locus, the genome is wrapped around by a donut-shaped ND10 body [105]. Although current technologies have not been able to detect the chromatin status of HSV genome in a single neuron, it is plausible to postulate that ND10 components colocalized to the latent HSV chromatin may regulate the intricate balance between latency and reactivation. Interestingly, in a cell culture model of quiescent HSV-1 infection, superinfection by an ICP0 RING finger mutant virus, which was incapable of degrading PML and Sp100 and therefore incapable of dispersing ND10, did not remove heterochromatin markers from the quiescent HSV-1 genome, whereas the wild type counterpart reduced the heterochromatin markers on HSV-1 genome and reactivated the quiescent genome into productive infection [24]. A recent report in the HIV research showed that latent HIV-1 proviruses are also in the close vicinity of ND10 loci in CD4+ T cells, with PML binding to the latent HIV-1 promoter and forming facultative heterochromatin at HIV genome. In addition, PML degradation and ND10 dispersal lead to the loss of heterochromatin marker and the reactivation of HIV-1 transcription [106]. Based on these findings, it is quite reasonable to hypothesize that in latent infection ND10 may also repress the HSV genome by reprogramming the HSV chromatin.

In lytic infection, the incoming HSV genome is found in the vicinity of ND10. Later on, viral replication compartments establish at the original ND10 loci after the dispersal of ND10 [72, 107, 108]. Evidence to show that ND10 components directly modulate the partial HSV nucleosomes in early lytic infection is still lacking. However, various experiments have indicated a potential link between ND10 repression and chromatin remodeling in the lytic infection. First, from the virus side, two HSV-1 proteins have been found to attack ND10 and regulate histone modification simultaneously. One is the aforementioned ICP0, which uses its RING type E3 ubiquitin ligase to degrade ND10 organizers and in the meantime interacts with CoREST to dislodge HDACs from the REST/CoREST/LSD1/HDAC complex [26, 65, 79]. The two ICP0 functions are interconnected with each other. The D671A/E673A substitutions that knocks out the CoREST binding also negatively affect the degradation of PML in the infected cells [23]. The second viral protein regulating both ND10 structure and chromatin remodeling is the γ1 (leaky late) product U_S_3. U_S_3 is a viral serine/threonine kinase that phosphorylates various cellular proteins to block apoptosis during infection [109]. One class of the proteins phosphorylated by U_S_3 are HDACs, including HDAC1 and HDAC2 [110, 111]. HDAC phosphorylation affects its interactions to its binding partners, such as CoREST, and therefore regulates gene expression in signal transduction and cell cycle control [112]. Surprisingly, overexpression of U_S_3 alone disrupts ND10 in transfected cells [113], suggesting that U_S_3 may interact with some ND10 components. In infected cells the time when U_S_3 starts to express (3–6 h post infection) overlaps with the time when degradation of PML and Sp100 is nearly completed. Whether U_S_3 participates in dispersing ND10 components has not been investigated. It will be interesting to see if U_S_3 phosphorylating HDAC and changing HSV chromatin status are related to the ND10 dispersal.

The second notable connection between ND10 and chromatin in HSV infection is the recruitment of various cellular chromatin regulators into the ND10 bodies. These proteins include the foreign DNA sensor IFI16, the corepressor protein CoREST, and a histone acetyltransferase CLOCK [108, 114, 115]. IFI16 was another repressive protein found to directly associate with the incoming HSV genome as early as 1 h post infection. This association caused significant chromatin repression of viral transcription whereas depletion of IFI16 released the repression [116]. The aforementioned CoREST being accumulated at ND10 was only observed in cells infected by ICP0-null virus, suggesting that ICP0-CoREST-ND10 interactions are likely in dynamics. Later in HSV-1 infection the aggregated CoREST was also found as part of the replication compartment [108], which may be in line with the fact that LSD1, the demethylase tightly bound to CoREST [117], is required for the HSV-1 replication [28]. The CLOCK protein recruited to ND10 was found beneficial for HSV-1 replication. The protein was stabilized during the infection. Overexpression of CLOCK promoted viral protein expression whereas depletion of the protein significantly decreased viral protein expression [114], suggesting the participation of CLOCK in HSV-1 gene activation.

So far, ND10 has been mainly viewed as a part of host anti-viral defense. However, ND10 also contains many gene activators and in reality it did activate CMV promoter-driven luciferase tethered to ND10 [103]. Therefore, it is plausible to postulate that HSV may adopt some positive factors located within ND10 in order to establish its replication compartments, which are initiated at the original ND10 loci [107, 108]. The recruitment of CoREST and CLOCK to ND10 and their involvement in viral replication and gene activation strongly supports this hypothesis. Interestingly, the recruitment of both CoREST and CLOCK involves their interactions with ICP0 protein, directly or indirectly [22, 114, 118], suggesting that ICP0 plays a critical role in viral replication not only by disrupting the ND10 structure to alleviate restriction but also by capturing useful ND10 components to stimulate replication.

## Conclusions

Epigenetic regulation and ND10 dynamic organization are two important aspects of host anti-viral defense mechanisms. Between the two, ND10, with its massive amount of component proteins moving in and out under different conditions, may participate in and modulate the chromatin remodeling process of HSV genome. Very likely, this dynamic nuclear structure acts as a molecular hub that both virus and host try to exploit the various components for their own benefits. On one hand, the host intends to bring the ND10 repressive components to HSV genome and modulate the partial HSV nucleosomes for chromatin repression. On the other hand, being one of the best evolutionarily adapted viruses in human history, HSV may use its multifunctional proteins such as ICP0 to take advantage of this molecular hub. It may try to recruit beneficial host proteins to the spot while repelling the restrictive factors by destroying ND10. Understanding the mechanisms of the coordination between different host defensive pathways and the interaction of various viral countermeasures to these host pathways is the key to solving the mysteries in herpes viral infection.

## Abbreviations

APL: acute promyelocytic leukemia
ATRX: α-Thalassemia/Mental Retardation Syndrome X-Linked
Daxx: death-domain associated protein
HCF-1: host cell factor 1
HCMV: human cytomegalovirus
HDAC: histone deacetylase
HP1: heterochromatin protein 1
HSV-1: Herpes Simplex Virus-1
ICP0: infected cell protein 0
IFN: interferon
LAT: latency-associated transcript
ND10: Nuclear Domain 10
PML-RARα: PML-retinoic acid receptor α
PRC: polycomb repressor complex
RA: retinoic acid
